# Complete chloroplast genome sequence of *Salix sinopurpurea* (Salicaceae)

**DOI:** 10.1080/23802359.2020.1858726

**Published:** 2021-03-11

**Authors:** Feiyi Guo, Kangjia Liu, Yachao Wang, Enze Li, Zhenfeng Zhan, Zhixiang Zhang

**Affiliations:** a School of Ecology and Nature Conservation, Beijing Forestry University, Beijing, China; b Museum of Beijing Forestry University, Beijing Forestry University, Beijing, China

**Keywords:** *Salix sinopurpurea*, complete chloroplast genome, phylogeny

## Abstract

*Salix sinopurpurea* is a morphologically special shrubby willow characterizing opposite leaves. Here, we reported the complete chloroplast (cp) genome sequence of *Salix sinopurpurea*. The cp genome is 155,546 bp in length, including a large single-copy (LSC) region of 84,412 bp, a small single-copy (SSC) region of 16,216 bp, and a pair of inverted repeated regions of 27,459 bp. The cp genome of *Salix sinopurpurea* encodes 130 genes, including 85 protein-coding genes, 37 tRNA genes, and eight rRNA genes. Phylogenetic tree showed that *Salix sinopurpurea* is closely related to *Salix psammophila* and *Salix suchowensis*.

*Salix sinopurpurea* C. Wang et C. Y. Yang is a shrubby willow that is always confused with *S. purpurea* L., *S. linearistipularis* (Franch.) Hao and *S. suchowensis* Cheng based on morphological features. *Salix sinopurpurea* is one of the unique species characterizing opposite leaves in genus *Salix*. Thus, *S. sinopurpurea* is an important species to shed light on the evolutionary and phylogenetic relation of genus *Salix*. In this study, we assembled and annotated the complete chloroplast (cp) genome of *S. sinopurpurea* (GenBank accession number: MW077725) to provide genomic and genetic sources for further research.

The fresh leaves of *S. sinopurpurea* were collected from Xian, Shaanxi province, China (N 108°48′49″, E 33°51′6″). The voucher specimens (collection number: HLCS19_18) were deposited in the Herbarium of Beijing Forestry University (BJFC). Genomic DNAs were extracted using CTAB method (Doyle and Doyle 1987), and 2 × 150 bp pair-end sequencing was performed on an Illumina HiSeq 4000 platform at Novogene (http://www.novogene.com, Beijing, China). The whole assembly process was conducted with Geneious 10.2 (Kearse et al. 2012), mainly following He et al. (2019), using *S. babylonica* (MF189167) as reference. The initial annotations of complete cp genome were performed using Plastid Genome Annotator (PGA) (Qu et al. 2019) with *Amborella trichopoda* (AJ506156) as reference, and verified manually using Unix program Plann 1.1.2 (Huang and Cronk 2015) and Geneious 10.2 (Kearse et al. 2012).

The cp genome of *S. sinopurpurea* was 155,546 bp in length, containing a large single-copy (LSC) region of 84,412 bp, a small single-copy (SSC) region of 16,216 bp, and a pair of inverted repeat (IR) regions of 27,459 bp. Genome annotation predicted 130 genes, including 85 protein-coding genes, 37 tRNA genes, and eight rRNA genes. The overall GC-content of the cp genome was 36.7%, while the corresponding values in the LSC, SSC, and IR regions were 34.4%, 31.0%, and 41.9%, respectively.

Phylogenetic inference suggested that *S. sinopurpurea* is closely related to *S. psammophila* and *S. suchowensis* (Figure 1), which was conducted using the best-fit model K3Pu + F+R2 according to Bayesian Information Criterion in IQ-TREE 1.6.12 (Nguyen 2015) with 5000 bootstrap replicates. The 23 complete cp genomes were aligned using MAFFT (Katoh and Standley 2013). This study could lay a foundation for species delimitation and evolutionary relation of genus *Salix* in the future.

**Figure 1.**
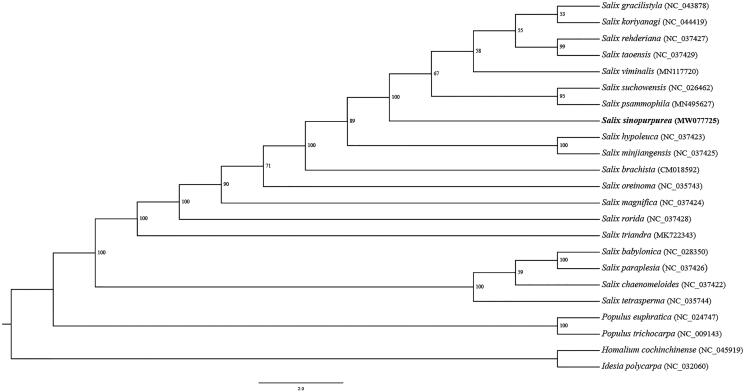
Maximum likelihood tree with 5000 bootstrap replicates constructed using IQ-TREE based on chloroplast genomes of 23 Salicaceae species.

## Data Availability

The data that support the findings of this study are openly available in GenBank of NCBI at https://www.ncbi.nlm.nih.gov/, reference number MW077725.
